# Implementation and evaluation of Pressurized IntraThoracic Aerosol Chemotherapy (PITAC) for the treatment of patients with malignant pleural effusion: study protocol for the Danish phase-I PITAC-OPC5 study

**DOI:** 10.1515/pp-2024-0014

**Published:** 2024-11-18

**Authors:** Pernille Schjødt Hansen, Martin Graversen, Sönke Detlefsen, Alan Patrick Ainsworth, Claus Wilki Fristrup, Lise Eckhoff, Mia Jelin-Klaric, Michael Bau Mortensen

**Affiliations:** Odense PIPAC Center (OPC) and Odense Pancreas Center (OPAC), Odense University Hospital, Odense, Denmark; Department of Surgery, HPB and Upper GI Section, Odense University Hospital, Odense, Denmark; Department of Pathology, Odense University Hospital, Odense, Denmark; Department of Oncology, Odense University Hospital, Odense, Denmark; Faculty of Health Sciences, Institute of Clinical Research, University of Southern Denmark, Odense, Denmark

**Keywords:** cancer-directed therapy, malignant pleural effusion, palliative treatment, pressurized intrathoracic aerosol chemotherapy, thoracic regression grading score

## Abstract

**Objectives:**

Pressurized IntraThoracic Aerosol Chemotherapy (PITAC) is a minimally invasive cancer-directed therapy for patients with malignant pleural effusion (MPE) and/or pleural metastasis (PLM). PITAC is based on Pressurized IntraPeritoneal Aerosol Chemotherapy, which has proven to be safe and feasible. Since 2012, 47 PITACs have been published, and prospective data on feasibility, safety and potential local response are lacking.

**Methods:**

The prospective, controlled, phase-I study is designed to treat MPE with PITAC. There are no data to support the estimated number of patients needed, but previous experience estimates the non-access rate to 20 %. Twenty eligible patients with MPE will receive two or more PITACs at four-week intervals. During video-assisted thoracoscopy, MPE and/or pleural lavage fluid is evacuated, and the extent of visible PLM is assessed. Pleural biopsies are collected, if possible, for histological response as per Thoracic Regression Grading Score (TRGS). Patients are screened for treatment-related intra- and postoperative complications. The primary outcome is the number of patients with Clavien-Dindo ≥3b or Common Terminology Criteria for Adverse Events≥4 within 30 days. Secondary objectives include PLM-score, TRGS and cytology, length of hospitalization, personnel safety, quality of life, and change in MPE volume.

**Results:**

PITAC is expected to be safe and feasible for patients and personnel, and achieve positive results in the reduction of MPE volume.

**Conclusions:**

The results may significantly impact the next clinical, technical, and scientific steps in the implementation of PITAC. Given the suboptimal treatment options for MPE and the seemingly promising results of PITAC, we find the implementation of PITAC ethically reasonable and sound.

## Introduction

When the spread of cancerous cells to the pleural cavity disrupts the equilibrium between absorption and excretion of fluid, the result is malignant pleural effusion (MPE) [1, 2]. Mesothelioma, lymphoma, lung, breast, ovarian, and gastrointestinal cancers are the most frequent causes of MPE [3]. MPE indicates an advanced disease with high mortality, poor quality of life and a life expectancy of 3–12 months [1, 4, 5]. The patients present with progressive shortness of breath, chest pain, cough and weight loss [5]. Recent research has suggested that MPE furthermore promotes tumour growth and chemotherapy resistance [6]. The exact incidence of MPE in Denmark is unknown, but is estimated to approximately 5,000 new cases per year and found equaling 15 % of all cancer patients [5, 7].

The available treatments for symptomatic MPE are suboptimal and with limited influence on patient’s life expectancy as these interventions focus only on relief of symptoms, and 90 % of the patients require repeated interventions [8, 9]. They include intermittent pleural drainage, indwelling pleural catheters, and chemical pleurodesis [5, 10]. Pleurodesis has shown a potential increase in survival suggesting that local treatment of the pleura should be prioritized [11].

Patients with MPE who are in a good general condition and with a remaining life expectancy of more than a few months may have an unmet need for additional treatment, in order to increase their quality of life [9]. Often, these patients have developed loculations, hindering complete drainage of MPE and undergone several different treatments with disappointing results. Hence, new and more effective treatment strategies are needed.

### Pressurized IntraThoracic Aerosol Chemotherapy (PITAC)

The first treatment with PITAC for MPE was performed in Germany in 2012. The concept of PITAC is based on experience from Pressurized IntraPeritoneal Aerosol Chemotherapy (PIPAC) [3]. PIPAC is feasible and safe in the treatment of peritoneal metastasis (PM) 12], [13], [14. In addition, PIPAC directed therapy is repeatable with 4–6 week intervals, and the patients may be discharged at postoperative day 0–1 without significant toxicity [13]. The intraperitoneal nebulization of chemotherapy under high pressure increases distribution and penetration of cytotoxic agents to the peritoneum, which may decrease fluid production 15], [16], [17. Theoretically, it should be possible to achieve the same effects in the intrapleural space when using PITAC, but whether this is feasible, safe and able to provide local response is uncertain [18].

To date, data on 21 patients treated with 38 PITACs have been published worldwide, and at Odense PIPAC Center (OPC), five patients have been treated with 11 PITAC procedures (two non-access). The primary indications were MPE and/or pleural metastasis (PLM) due to malignant mesothelioma, gastric cancer, ovarian cancer, rectal cancer, or breast cancer. A recent review by Hansen et al. suggests that PITAC is feasible, but more prospective data are needed to evaluate patient and personnel safety, and potential local effect [3, 15, 19], [20], [21], [22.

### Aim

This study aims to evaluate patient and personnel safety during the implementation of PITAC in Denmark.

#### Primary and secondary objectives

This is a Phase-I study investigating safety and feasibility of repeated PITAC (minimum of two procedures) in 20 patients. PITAC is considered feasible and safe if four patients or less have severe surgical complications defined as Clavien-Dindo ≥ 3b or life-threatening adverse events (AE) defined as Common Terminology Criteria for Adverse Events v. 6.0 (CTCAE) ≥ 4 within 30 days after the procedure. All medical AEs and surgical complications will be recorded, but only those who are probably or certainly/definitely related to the PITAC directed treatment will provide grounds for assessing the feasibility and safety.

Secondary objectives include (1) macroscopic evaluation of PLM before and after PITAC and evaluation of histological response using a proposed Thoracic Regression Grading Score (TRGS) in biopsies from visible PLM and MPE [23], (2) evaluation of response by pleural lavage fluid (PLF) or MPE cytology, (3) assessment of personnel safety (environmental and biological), and (4) evaluation of the quality of life with EORTC-QLQ-C30 at baseline, day 30 and three months after the last PITAC directed treatment.

## Methods

### Recruitment process

Patients with MPE from any primary tumour visible with bedside ultrasound and drained at least one time are eligible for inclusion (Table 1). Patients with MPE who are eligible for PITAC are identified during the multidisciplinary tumour (MDT) conference. Bilateral MPE is acceptable, but the most affected side at the time of inclusion will be treated throughout this study. In case of symptomatic MPE on the contralateral side, the patient may be included as a new patient after the last follow-up.

**Table 1:** Inclusion and exclusion criteria.

Inclusion criteria	Exclusion criteria
– Symptomatic MPE visible with bedside ultrasound – Histologically or cytologically verified malignancy (primary tumour) – Status CT-scan not older than four weeks – MPE requiring at least one drainage procedure – Drained ≥14 days before the first PITAC directed treatment – Bidirectional systemic chemotherapy or immunotherapy ≥14 days before the first PITAC directed treatment or no simultaneous systemic chemotherapy or immunotherapy – ECOG performance status 0-2 – Life expectancy ≥3 months – Age ≥18 years – Fertile women must have a negative pregnancy test at time of inclusion and use adequate contraception – Danish-speaking and reading patients	– A history of allergic reaction to cisplatin or other platinum containing compounds or doxorubicin – Renal impairment, defined as GFR<40 mL/min (cockcroft-gault equation) – Myocardial insufficiency, defined as NYHA class>2 – Impaired liver function defined as bilirubin≥1.5*xULN* – Impaired bone marrow function defined as platelets≤100*x*10^9^/L – Any other condition or therapy, which in the investigator´s opinion may pose a risk to the patient or interfere with the study objects

CT, computed tomography; ECOG, eastern cooperative oncology group; GFR, glomerular filtration rate; MPE, malignant pleural effusion; NYHA, New York heart association; PITAC, pressurized intrathoracic aerosol chemotherapy; PLM, pleural metastasis; ULN, upper limit of normal.

All personnel will be informed about the procedure both orally and in writing, including occupational health aspects. Only those who volunteer will take part in the PITAC procedure.

Patients will be excluded from the study if (1) requested by the patient, (2) access to the pleural cavity is not possible due to adhesions or other technical aspects, and/or (3) decline in general health resulting in Eastern Cooperative Oncology Group (ECOG) performance status higher than 2. Following events are considered sufficient reasons for discontinuing a subject from the study (1) withdrawal of consent, (2) death, (3) loss to follow up and/or (4) severe AE or surgical complications. The following information will be recorded for patients who have been excluded from the study: Survival, amount and frequency of MPE drainages and illness specific variables (e.g. CT-scan results).

### Study intervention and procedures

**Figure 1:**
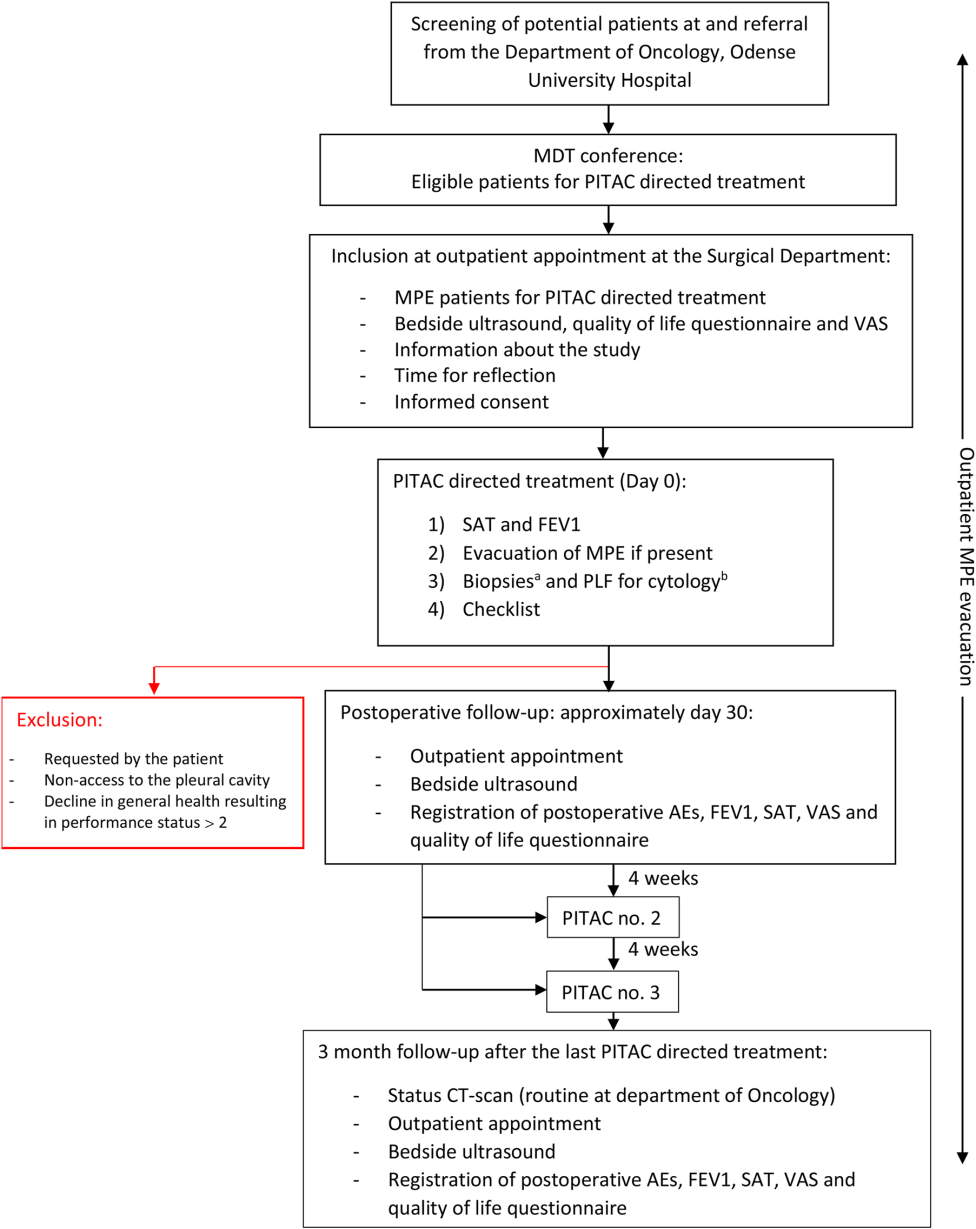
Study flow chart ^a^if visible PLM; ^b^if less than 150 mL of MPE present. AEs: adverse events; FEV1: forced expired volume in the first second; MDT: multidisciplinary tumour; MPE: malignant pleural effusion; PITAC: pressurized intrathoracic aerosol chemotherapy; PLF: pleural lavage fluid; PLM: pleural metastasis; SAT: saturation; TRGS: thoracic regression grading score; VAS: visual analogue score.

The patients will be scheduled for clinical assessment in the PITAC outpatient clinic including bedside ultrasound of the pleural cavity and information about the study. Included patients will be scheduled for a maximum of three PITAC procedures and minimum of two PITAC procedures at four-week intervals (Figure 1).

Patients may have a pleurocentesis up to 14 days before the planned PITAC directed treatment. In case the patients require drainage of MPE between PITACs, this is possible as an outpatient procedure and registered to ensure that the accumulative amount of MPE from the first PITAC directed treatment can be calculated. Patients may receive treatment with systemic chemotherapy or immunotherapy up to 14 days before PITAC treatment, and antihormonal, tyrosine kinase inhibitor (TKI) and/or steroid treatment may be given continuously. The wash out period from PITAC to systemic chemotherapy or immunotherapy is one week.

### Preoperative procedure

Baseline blood samples are taken two days before each PITAC. Prophylactic low molecular weight heparin is given before and after the procedure. Oxygen saturation (SAT), forced expired volume in the first second (FEV1), and visual analogue scale (VAS) pain and breathlessness will be measured before each PITAC.

### PITAC

The procedure will be performed in an operating room (OR) with a modern direct flow ventilation system that complies with ISO norm class 5. The chemotherapy is evacuated through a closed air waste system at the end of the procedure. The operating personnel will wear protective barrier garments, double layered gloves and glasses throughout the entire procedure.

**Figure 2:**
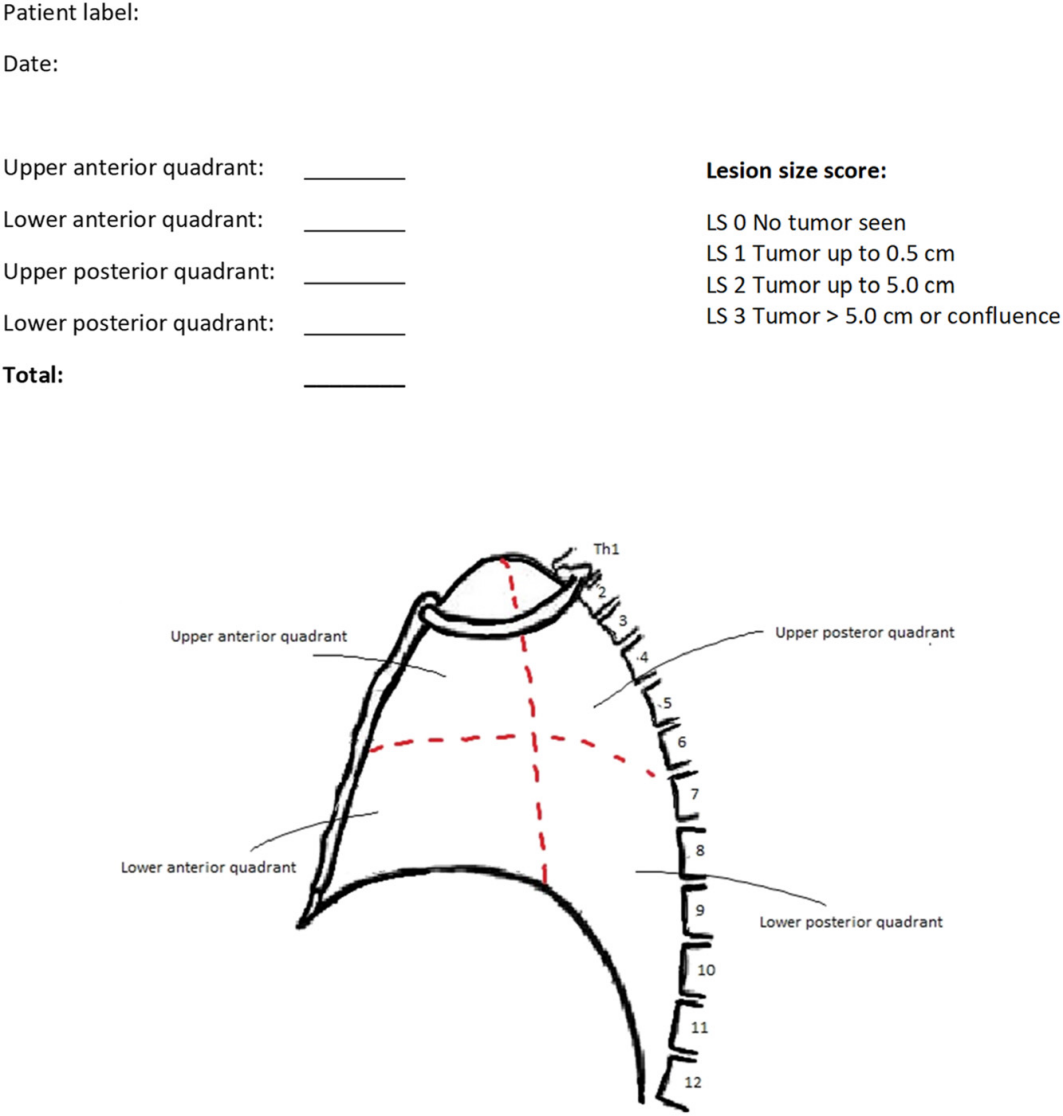
Macroscopic evaluation of visible pleural metastasis (PLM) (PLM-score).

Prior to general anaesthesia a bedside ultrasound is performed in the OR to ensure the presence of MPE. General anaesthesia and prophylactic antibiotics are administered and the patient is intubated in the supine position and flipped into the prone position. In the prone position ultrasonography is repeated to ensure optimal access to the pleural cavity, and the first entry point is marked with a pen before surgical dressing is applied. Under video-assisted thoracoscopy two trocars (5 and 12 mm) are placed and exclusion of the ipsilateral lung is confirmed when relevant. MPE is removed (and submitted for cytology) and measured and the extent of visible PLM is assessed macroscopically using the PLM-score (Figure 2). If no or only small amounts of MPE are present, pleural lavage is performed and PLF is submitted for cytology. The PLM-score is a modification of the known PCI-score. The parietal pleura is divided into four quadrants and assessed on a scale from 0-3 depending on the size of visible tumour lesions (Figure 2). Quadrant biopsies are taken from visible PLM on the parietal pleura for histological assessment.

The CE-certified nebulizer is inserted pointing away from lung tissue. A safety checklist is followed to ensure maximum safety for both patients and personnel.

PITAC is performed at an intrapleural pressure of 12 mmHg using normothermic carbon dioxide (CO_2_). The injector is controlled from outside the OR and the settings will be a maximum of 300 pound-force per square inch (Psi) with a flow-rate of 0.5–1.8 mL/s as defined for the used nebulizer. Diffusion time is 30 min. The injector and patient observation monitors are visible through a window.

The chemotherapy filled air in the pleural cavity is removed through a closed air waste system. Sufficient re-ventilation of the ipsilateral lung is ensured when relevant/possible. The two trocar access points are closed with suture in both the fascia and skin. Chest tubes are not used routinely. Local anaesthetics are applied at the trocar sites.

### Histology

Quadrant parietal pleural biopsies will be collected, if possible. The biopsies will be fixed in formalin and embedded in paraffin followed by step-section at three levels, and stained with haematoxylin and eosin (H&E), followed by a section immunostained for epithelial cell adhesion molecule (Ep-CAM (or Hector Battifora and MEsothelioma 1, HBME1, in case of malignant mesothelioma)), and a final series of three step sections stained with H&E [24, 25]. Additional immunostains will be used at the discretion of the pathologist. Histological response to treatment will be assessed in each biopsy according to the proposed TRGS, a modification of the Peritoneal Regression Grading Score (PRGS) [23, 26, 27]. TRGS-1 equals complete histological response, and is defined as the absence of residual cancer and harbours only regressive histological features. TRGS-2 is defined as a biopsy where regressive changes are predominant over cancer cells (major response). TRGS-3 is a biopsy where cancer cells are predominant but regressive changes are present (minor response), and TRGS-4 is a biopsy with cancer but without histological features of regression (no response).

### Cytology

A maximum of 250 mL MPE or PLF pr. PITAC procedure will be collected and analysed. Pleural lavage is performed in cases with no or less than 200 mL of MPE present in the pleural space. The fluid will be centrifuged, and smears of the sediment analysed by conventional cytology (Papanicolaou and May-Giemsa Grünwald staining). Leftovers of the sediment will be embedded in paraffin wax of which one section will be stained with H&E. If necessary, further sections will as part of the standard routine be cut for immunocytochemical analyses for markers such as calretinin, thyroid transcription factor 1 (TTF1), carcinoembryonic antigen (CEA), cytokeratin (CK) 7, CK20, synaptophysin, cluster of differentiation (CD) 56 or P40. The cytological specimens will be categorized as one of the following: Malignant cells, cells suspicious of malignancy, atypical cells, no malignant cells, or ”inconclusive” (for technical reasons).

### Chemotherapy

Depending on the primary tumour and previous/bidirectional systemic chemotherapy, the following regimes will be applied: Patients with MPE from non-colorectal or -appendix cancer will be treated with cisplatin (10.5 mg/m^2^ body surface in 150 mL saline) and doxorubicin (2.1 mg/m^2^ body surface in 50 mL saline). Patients with MPE from colorectal or appendiceal cancer will receive oxaliplatin (92 mg/m^2^ body surface in 150 mL dextrose).

Cisplatin, oxaliplatin and doxorubicin are standard, commercially available cytostatic drugs in oncologic treatment with alkylating and topoisomerase inhibitor effect, respectively. The most common side effects of administering Cisplatin and oxaliplatin intravenously are: Fever, nausea, diarrhoea, kidney failure, pancytopenia and ototoxicity. With regard to doxorubicin, it is furthermore: Debilitation, shivers, infections, skin eruptions, electrocardiogram changes, thrombophlebitis and deep venous thrombosis. Based on the literature and experience gained from more than 1000 PIPAC procedures at OPC, PIPAC (i.e. local treatment) with cisplatin, oxaliplatin and doxorubicin is well tolerated with only a minimal of nausea, vomiting and transient abdominal pain. The experience with PITAC directed therapy is limited, but the above mentioned systemic adverse events from cisplatin, oxaliplatin and doxorubicin are not expected with PITAC directed treatment. The use of cisplatin 10.5 mg/m^2^, oxaliplatin 92 mg/m^2^ and doxorubicin 2.1 mg/m^2^ are based on dose finding studies, stating that the above-mentioned dosages are safe for intraperitoneal use [28, 29].

### Postoperative procedure

The patient is monitored for a minimum of one day. A chest X-ray is performed if the patient shows signs of respiratory distress to consider the indication for a chest tube. SAT, FEV1, and reactions/complications will be registered before discharge of the patient.

Thirty days after each PITAC directed therapy the patient will attend an outpatient appointment for bedside ultrasound and registration of postoperative reactions/complications. The 30 day follow-up must be collected prior to the next PITAC directed treatment.

The last follow-up including a CT scheduled three months after the last PITAC. Furthermore, patients will be evaluated in the outpatient clinic including bedside ultrasound, SAT and FEV1.

### Occupational health safety aspects

Summarized, there are three barriers between personnel and the chemotherapy: The closed intrathoracic cavity, the modern OR ventilation with a direct air-flow system, and the OR walls. Possible air-leakage is controlled by allowing a maximum of CO_2_ flow of 0.1–0.2 L/min corresponding to the intraperitoneal body resorption of CO_2_. The intrapleural air saturated with chemotherapy is evacuated through a series of filters into a closed ventilation system. Special chemotherapy waste bins are available for all single-use equipment and the trocar sites, removal and disposal of dressings and syringes are performed by the surgeon alone.

From previous PIPAC knowledge, is has been demonstrated that the procedure is safe in terms of the personnel safety and fulfils the European Community working space law. It is theorized that these data can be directly transferred to the PITAC procedure [12, 14]. Danish Technological Institute will analyse the toxicological workspace for traces of platinum to validate the occupational health safety during PITAC.

In the present study, blood samples from the surgeons will be analysed for traces of platinum immediately after the first procedure and again after the 10th procedure. The surgeons also perform weekly PIPAC procedures. Previous tests for platinum in the surgeońs blood after PIPAC were negative, why we expect only negative results [12].

### Study design

This is a non-randomized, non-blinded, prospective, single-center phase-I study designed to treat patients with MPE from any primary tumour with PITAC.

### Sample size

There are no data to support the estimated number of patients needed for this phase-I study. The non-access rate, based on previous experience, is estimated to 20 %. This study will include consecutive MPE patients until 20 patients have completed at least two PITAC procedures. Interim analyses will be performed in order to monitor patient safety.

### Ethical declarations

The study will be conducted in compliance with the protocol, principles of good clinical practice (ICH-GCP), Regulation (EU) No 536/2014, and in accordance with the ethical principles put forward in the second Declaration of Helsinki. The study is GCP monitored and has been approved by the Danish Medicines Agency and Ethical Committee (EU CT number 2023-503297-20-00). Oral and written consent from patients is mandatory and collected before any study-related procedures are performed.

### Statistics

Demographic data will be presented with range and median where appropriate.

#### Interim analysis

The reported risk of serious complications during standard thoracoscopy is approximately 5 % [30, 31]. Based on our preliminary experience with PITAC and the limited data from the literature, the risk of adding PITAC to a standard thoracoscopy seems low. However, since the actual risk of PITAC is unknown, we choose to perform an interim safety analysis after every 10 procedures. The study will be discontinued if four or more procedures have led to patients having Clavien-Dindo ≥ 3b or CTCAE≥4 complications/adverse events.

## Results and discussion

### Hypothesis

Based on OPC´s experience with PIPAC and preliminary data from previously performed PITAC procedures, we expect that this study will show that PITAC is both feasible and safe for patients and the involved health care personnel.

## References

[1] Asciak R, Rahman NM (2018). Malignant pleural effusion: from diagnostics to therapeutics. Clin Chest Med.

[2] Semaan R, Feller-Kopman D, Slatore C, Sockrider M (2016). Malignant pleural effusions. Am J Respir Crit Care Med.

[3] Jonscher N, Hummels M, Giger-Pabst U, Karljalainen E, Zieren J, Büchner N, Raymond MA (2014). Chapter 18 pressurized IntraThoracic aerosol chemotherapy (PITAC). The book of PIPAC – cancer under pressure.

[4] Egan AM, McPhillips D, Sarkar S, Breen DP (2014). Malignant pleural effusion. QJM.

[5] Fjællegaard K, Petersen JK, Armbuster K, Jensen HK, Skaarup SH, Laursen CB (2021). Malign pleuraeffusion. Ugeskr Laeger.

[6] Cheah HM, Lansley SM, Varano Della Vergiliana JF, Tan AL, Thomas R, Leong SL (2017). Malignant pleural fluid from mesothelioma has potent biological activities. Respirol.

[7] Dipper A, Jones HE, Bhatnagar R, Preston NJ, Maskell N, Clive AO (2020). Interventions for the management of malignant pleural effusions: a network meta-analysis. Cochrane Database Syst Rev.

[8] Ferreiro L, Suárez-Antelo J, Álvarez-Dobaño JM, Toubes ME, Riveiro V, Valdés L (2020). Malignant pleural effusion: diagnosis and management. Can Respir J.

[9] Sivakumar P, Saigal A, Ahmed L (2020). Quality of life after interventions for malignant pleural effusions: a systematic review. BMJ Support Palliat Care.

[10] Bashour SI, Mankidy BJ, Lazarus DR (2022). Update on the diagnosis and management of malignant pleural effusions. Respir Med.

[11] Hassan M, Harriss E, Mercer RM, Rahman NM (2021). Survival and pleurodesis outcome in patients with malignant pleural effusion – a systematic review. Pleura Peritoneum.

[12] Graversen M, Pedersen PB, Mortensen MB (2016). Environmental safety during the administration of pressurized IntraPeritoneal aerosol chemotherapy (PIPAC). Pleura Peritoneum.

[13] Blanco A, Giger-Pabst U, Solass W, Zieren J, Reymond MA (2013). Renal and hepatic toxicities after pressurized intraperitoneal aerosol chemotherapy (PIPAC). Ann Surg Oncol.

[14] Solass W, Giger-Pabst U, Zieren J, Reymond MA (2013). Pressurized intraperitoneal aerosol chemotherapy (PIPAC): occupational health and safety aspects. Ann Surg Oncol.

[15] Kuchen NCT, Hailemariam S, Schoeb O (2018). Safety and efficacy of pressurized intraperitoneal/intrathoracic aerosol chemotherapy (PIPAC/PITAC) in patients with peritoneal and/or pleural carcinomatosis: a preliminary experience. J Med Ther.

[16] Flessner MF (2005). The transport barrier in intraperitoneal therapy. Am J Physiol Renal Physiol.

[17] Mortensen MB, Casella F, Düzgün Ö, Glehen O, Hewett P, Hübner M (2023). Second annual report from the ISSPP PIPAC database. Pleura Peritoneum.

[18] Graversen M, Detlefsen S, Ainsworth AP, Fristrup CW, Knudsen AO, Pfeiffer P (2023). Treatment of peritoneal metastasis with pressurized intraperitoneal aerosol chemotherapy: results from the prospective PIPAC-OPC2 study. Ann Surg Oncol.

[19] Drevet G, Maury JM, Bakrin N, Tronc F (2020). Technique of pressurized intrathoracic aerosol chemotherapy (PITAC) for malignant pleural effusion. Pleura Peritoneum.

[20] Giger-Pabst U, Demtröder C, Falkenstein TA, Ouaissi M, Götze TO, Rezniczek GA (2018). Pressurized IntraPeritoneal aerosol chemotherapy (PIPAC) for the treatment of malignant mesothelioma. BMC Cancer.

[21] Robella M, Vaira M, Borsano A, Mossetti C, M DES (2018). Low-dose pressurized intrathoracic aerosol chemotherapy (PITAC) as an alternative therapy for pleuropulmonary involvement in pseudomyxoma peritonei. Anticancer Res.

[22] Hansen PS, Graversen M, Detlefsen S, Mortensen MB (2024). Review on treatment of pleural metastasis and malignant pleural effusion with Pressurized IntraThoracic Aerosol Chemotherapy (PITAC). Pleura Peritoneum.

[23] Hansen PS, Graversen M, Detlefsen S, Ainsworth AP, Mortensen MB (2024). Pressurized IntraThoracic Aerosol Chemotherapy (PITAC) directed therapy of patients with malignant pleural effusion and/or pleural metastasis. ..

[24] Graversen M, Detlefsen S, Bjerregaard JK, Fristrup CW, Pfeiffer P, Mortensen MB (2018). Prospective, single-center implementation and response evaluation of pressurized intraperitoneal aerosol chemotherapy (PIPAC) for peritoneal metastasis. Ther Adv Med Oncol.

[25] Detlefsen S, Windedal T, Bibeau F, Bruhn LV, Carr N, Graversen M (2022). Role of immunohistochemistry for interobserver agreement of Peritoneal Regression Grading Score in peritoneal metastasis. Hum Pathol.

[26] Solass W, Sempoux C, Detlefsen S, Carr NJ, Bibeau F (2016). Peritoneal sampling and histological assessment of therapeutic response in peritoneal metastasis: proposal of the Peritoneal Regression Grading Score (PRGS). Pleura Peritoneum.

[27] Solass W, Sempoux C, Carr NJ, Bibeau F, Neureiter D, Jäger T (2019). Reproducibility of the peritoneal regression grading score for assessment of response to therapy in peritoneal metastasis. Histopathology.

[28] Dumont F, Passot C, Raoul JL, Kepenekian V, Lelièvre B, Boisdron-Celle M (2020). A phase I dose-escalation study of oxaliplatin delivered via a laparoscopic approach using pressurised intraperitoneal aerosol chemotherapy for advanced peritoneal metastases of gastrointestinal tract cancers. Eur J Cancer.

[29] Tempfer CB, Giger-Pabst U, Seebacher V, Petersen M, Dogan A, Rezniczek GA (2018). A phase I, single-arm, open-label, dose escalation study of intraperitoneal cisplatin and doxorubicin in patients with recurrent ovarian cancer and peritoneal carcinomatosis. Gynecol Oncol.

[30] Metintas M, Ak G, Yildirim H, Danacioglu S, Dundar E, Metintas S (2013). The safety of medical thoracoscopy in a group at high risk for complications. J Bronchology Interv Pulmonol.

[31] Wan YY, Zhai CC, Lin XS, Yao ZH, Liu QH, Zhu L (2019). Safety and complications of medical thoracoscopy in the management of pleural diseases. BMC Pulm Med.

